# The ‘straight mouse’: defining anatomical axes in 3D embryo models

**DOI:** 10.1093/database/bax010

**Published:** 2017-03-11

**Authors:** Chris Armit, Bill Hill, S. Venkataraman, Kenneth McLeod, Albert Burger, Richard Baldock

**Affiliations:** 1 MRC Human Genetics Unit, Institute of Genetics and Molecular Medicine, University of Edinburgh College of Medicine and Veterinary Medicine, Edinburgh, Scotland EH4 2XU, UK and; 2 Department of Computer Science, Heriot-Watt University, Edinburgh, Scotland EH14 4AS, UK

## Abstract

A primary objective of the eMouseAtlas Project is to enable 3D spatial mapping of whole embryo gene expression data to capture complex 3D patterns for indexing, visualization, cross-comparison and analysis. For this we have developed a spatio-temporal framework based on 3D models of embryos at different stages of development coupled with an anatomical ontology. Here we introduce a method of defining coordinate axes that correspond to the anatomical or biologically relevant anterior–posterior (A–P), dorsal–ventral (D–V) and left–right (L–R) directions. These enable more sophisticated query and analysis of the data with biologically relevant associations, and provide novel data visualizations that can reveal patterns that are otherwise difficult to detect in the standard 3D coordinate space. These anatomical coordinates are defined using the concept of a ‘straight mouse-embryo’ within which the anatomical coordinates are Cartesian. The straight embryo model has been mapped via a complex non-linear transform onto the standard embryo model. We explore the utility of this anatomical coordinate system in elucidating the spatial relationship of spatially mapped embryonic ‘*Fibroblast growth factor*’ gene expression patterns, and we discuss the importance of this technology in summarizing complex multimodal mouse embryo image data from gene expression and anatomy studies.

**Database URL:** www.emouseatlas.org

## Introduction

The concept of using coordinate systems to understand embryo development has a long history and was pioneered by D’Arcy Thompson in his seminal work *On Growth and Form* (1). In this approach, spatial warping of Cartesian coordinate systems was used to illustrate morphological evolutionary changes that are readily observed in nature. More recently, polar coordinate systems have been used to define positional information in the developing and regenerating limb, and to accurately predict the incidence of supernumerary limb element formation in both vertebrates and insects (2, 3). In the adult murine brain coordinate systems (4) are used routinely to define position and direction but only for the extent of the brain tissue and cannot be used elsewhere. Despite the proven utility of coordinate systems in allowing us to record data such as injections sites or understand the developing and regenerating limb, there has not been an equivalent coordinate space introduced into 3D models of vertebrate embryo development. The reasons for this are inherent in the complexity of the developing embryo, with the mammalian embryo being particularly variable in visual presentation and pose.

A primary objective of the eMouseAtlas Project is to enable 3D spatial mapping of whole embryo gene expression patterns to allow complex 3D gene expression patterns to be visualized and cross-compared. The spatial mapping process used by the eMouseAtlas Gene Expression database (EMAGE) uses points of equivalence defined on a source gene-expression data image and a morphological stage-matched embryo model. These points are used to define a spatial transformation, which maps the assay image coordinate system onto the coordinate system of the reference model. Having defined the transform, the assay signal in the original image is extracted and a representation of the original pattern is defined within the space of a 3D atlas model. Mapping patterns in this way provides an objective and accurate representation of the whole gene expression patterns without constraining it to anatomical boundaries (ontology annotation) and additionally captures information on expression gradients and strength variation. In addition to the Euclidean space of the reference model and of the assay images mapped to it, each model in a biomedical spatial atlas will also have an implicit ‘anatomically based’ coordinate system.

This anatomical coordinate system, understood by anatomists and biologists, allows locations and directions to be specified in a manner relative to the structure of the organism and which is independent of the organism pose. In an effort to develop an anatomical, or natural coordinate system (5) for the mouse embryo, we have developed a straightened, idealized mouse embryo model that allows us to define the anterior–posterior (A–P), dorsal–ventral (D–V) and left–right (L–R) axes in the head, trunk, tail and limb regions of our 3D embryo models. The transformation required to map the image of an embryo in its normal configuration to a straightened embryo is necessarily complex, requiring very large displacements and deformations. Until recently it has not been possible to produce such transformations, however with the advent of constrained distance transforms (CDTS, 6) such transformations have now become possible.

CDT relies on having a segmented target model—in this case the straight embryo model—and a collection of landmarks identifying points of equivalence. To aid in identifying landmarks we used a mouse embryo with a *Sonic hedgehog* (*Shh*) gene expression pattern. This expression pattern accurately defines midline structures in the head, trunk and tail region (7) and shows restricted expression in the posterior region of the developing fore- and hind-limb buds. To build a straight embryo model we have used image processing operations—including digital re-sectioning and surface modelling—on a 3D image of a *Shh*-labelled mouse embryo. A CDT transformation was then used to straighten embryos from their natural, curled conformation. In the space of the straight mouse embryo the anatomical coordinates are Cartesian, with *xy*, *yz* and *zx* representing true anatomical ‘transverse’, ‘sagittal’ and ‘coronal’ planes in the 3D model. By mapping the anatomical coordinates, as defined on the straight mouse embryo model, back onto the original curved 3D model, the anatomical axes are defined at all locations in the original 3D model.

We have explored the use of this anatomical coordinate system in elucidating the spatial relationship of embryonic *Fibroblast growth factor* (*Fgf*) gene expression patterns. *Fgf* genes encode a family of growth factor ligands that are critically important in organogenesis, craniofacial development and neuronal differentiation and survival. To understand the spatial relationships that exist between *Fgf* gene family members we used the anatomical coordinates to determine the A–P, D–V and L–R distribution of *Fgf8*, *Fgf9*, *Fgf10* and *Fgf20* and display them in the Cartesian frame of the straight mouse.

In this article, we first describe the development of a straight mouse embryo model and then its use in the visualization of spatial data. We propose that visualization using a straightened embryo reduces ambiguity and results in increased clarity, which we demonstrate using anatomical domains and spatially mapped gene expression patterns of the *Fgf* gene family. We further demonstrate the utility of the straightened mouse in a concise graphical summary of multiple gene expression patterns.

## Materials and methods

### Developing the straight mouse embryo framework

**Figure 1.**
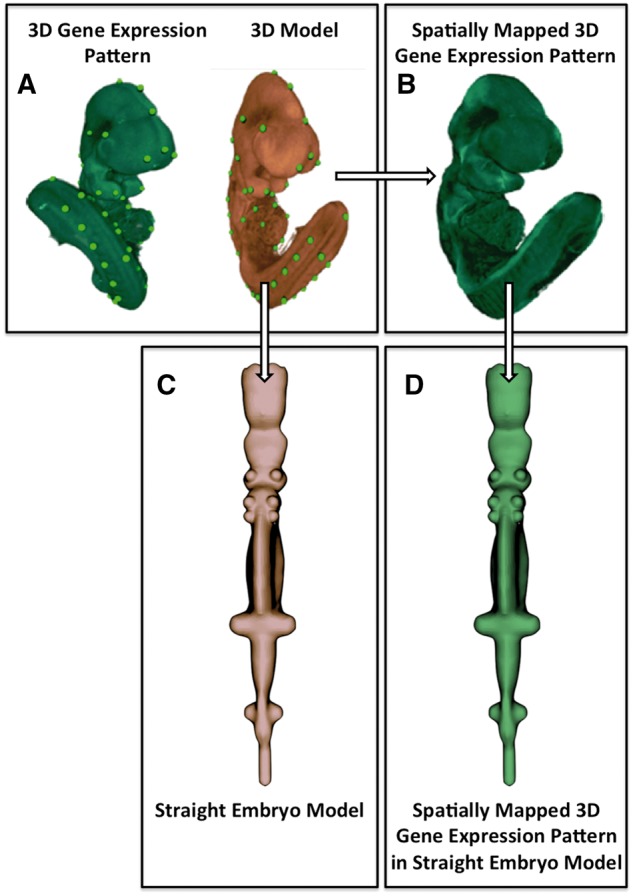
Image processing pipeline used to generate the straight mouse embryo. **(A)** Spatial warping using WlzWarp allows a user to place landmark points on a source image with gene expression (green) and a 3D model (orange) that utilizes a volumetric mesh. The green dots represent equivalent landmark points. **(B)** The spatial transform enables the gene expression pattern (green) to be mapped into the 3D model. Spatial warping of gene expression patterns using WlzWarp has been described previously in (10). **(C)** Spatial warping using WlzWarp was additionally used to straighten the mouse embryo model (landmark points not shown). The WlzWarp processing utilizes a CDT that enables complex non-linear deformations to be applied to 3D objects. (**D**) Gene expression patterns that were mapped into the 3D model (green) can be visualized in the context of the straight mouse embryo model.

The development of the straight mouse embryo framework comprised two steps: (i) the creation of a straight mouse embryo model from image data; and (ii) the creation of a transformation allowing a mapping between the anatomical coordinate system—as defined on the straight mouse embryo and the original 3D embryo model. An overview of the image data processing pipeline is shown in Figure 1.

### Generation of the straight mouse embryo model

A 3D Optical Projection Tomography (OPT; 8) image of a representative E10.5, Theiler stage 17 (TS; 9) CD1 mouse embryo, was colourimetrically labelled using alkaline phosphatase and NBT/BCIP to show the expression of *Shh*. This was used as the basis for the straight embryo model. *Shh* was selected as it is a signalling molecule with an expression pattern that accurately defines the midline structures in the head, trunk and tail regions of the mouse embryo at this stage (7).

A set of sagittal and coronal section images were digitally resectioned from the volumetric image using the image processing application MAPaint (freely available from https://github.com/ma-tech/) and assembled together with selected transverse plane images to form a model composed of orthogonal planes. Because of the high curvature of the embryo at TS17, particularly evident in the sagittal plane, the sampling was non-uniform with fewer samples in the ventral than dorsal regions. From these orthogonal planes an enclosing surface model was constructed using the 3D creation tool Blender (https://www.blender.org/) so as to smoothly interpolate between the embryo boundary segments. Because of the complex nature of the embryo outer boundary, portions of it were considerably simplified in building the straight embryo surface model. Principally, these were in the vicinity of the branchial arches, and additionally in ventral regions such as the heart and the genital tubercle.

When building the straight mouse surface model, care was taken to ensure that *Shh* expression in the neural tube, evident in the image planes, formed the z-axis of the model’s Cartesian coordinate system. This resulted in a straight embryo surface model in which the Cartesian planes represented the cardinal anatomical planes such that the *x-y*, *y-z* and *z-x* planes represented transverse, sagittal and coronal planes, respectively.

### Mapping the curved embryo to the straight embryo model

Corresponding landmark points were defined on the curved embryo OPT image and the volumetric straight embryo. Care was taken to place landmarks on embryo boundaries, particularly at points corresponding to orthogonal planes in regions of high curvature and at prominent features. The expression pattern of *Shh* was used to guide the placement of landmarks primarily along the A–P axis where it follows the embryo midline, and also in the fore- and hind-limb buds where it is expressed in the zone of polarizing activity (ZPA) i.e. the posterior region of the limb buds. Further landmarks were then added through distance subdivision until the surfaces and internal features were brought into register. To register the two models, a CDT (Hill and Baldock, 2015) was computed using landmark points and a conforming tetrahedral mesh that was generated from the straight embryo surface using Netgen (https://sourceforge.net/projects/netgen-mesher/). Because CDTs are invertible, the transformation defined allowed mapping both to and from the straight embryo model. It is these mappings that form the core of the straight mouse embryo framework since they map an anatomical, Cartesian coordinate system to the space of the original mouse embryo.

### Spatial mapping of gene expression data

To register gene expression data into the eMouseAtlas models, in this case *Fgfs* and *Shh* into our TS17 model, CDT was used through a graphical user interface, WlzWarp (10). This software allows user-defined placement of landmarks pair-wise at points of equivalence between a source (experimental data) and target (reference model). The resultant warped data can then be image processed, for instance in terms of threshold values to qualitatively reflect the levels of gene expression intensity and concomitantly exclude any artefacts.

## Results

### The ‘straight mouse’ embryo model accurately defines the A–P and D–V axes in 3D space

**Figure 2.**
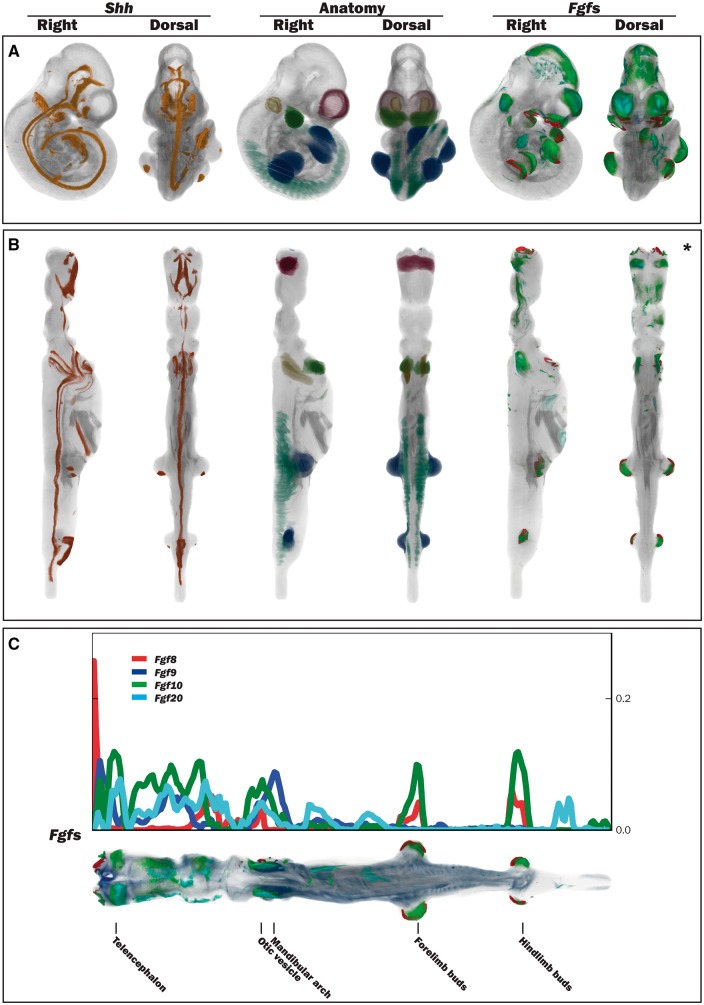
Straightening the 3D mouse embryo model. **(A)** Dorsal and lateral views of the original TS17 (E10.5) curled embryo model showing Shh expression, delineated anatomical components and Fgf gene expression. **(B)** Dorsal and lateral views of the respective straightened embryo model to that in A. Shh expression: observed in the A–P axis midline in the original and straight embryo models, and also in the posterior region of the forelimb and hindlimb buds. Anatomy domains: those shown are the telencephalic vesicles, otic vesicles, mandibular arches, forelimb and hindlimb buds and the somites. Fgf8, Fgf9, Fgf10 and Fgf20 expression patterns: these were spatially mapped as a test case to evaluate the utility of the straight mouse as a data visualization tool. **(C)** Graphical representation of Fgf gene expression patterns along the A–P axis in relation to the straight mouse embryo. The straight embryo shown is the same as that in B*, but with different opacity and thresholding values. Plot shows distribution of spatially mapped patterns along the A–P axis with the fraction of volume occupied by the thresholded gene expression patterns on the vertical axis.

**Figure 3.**
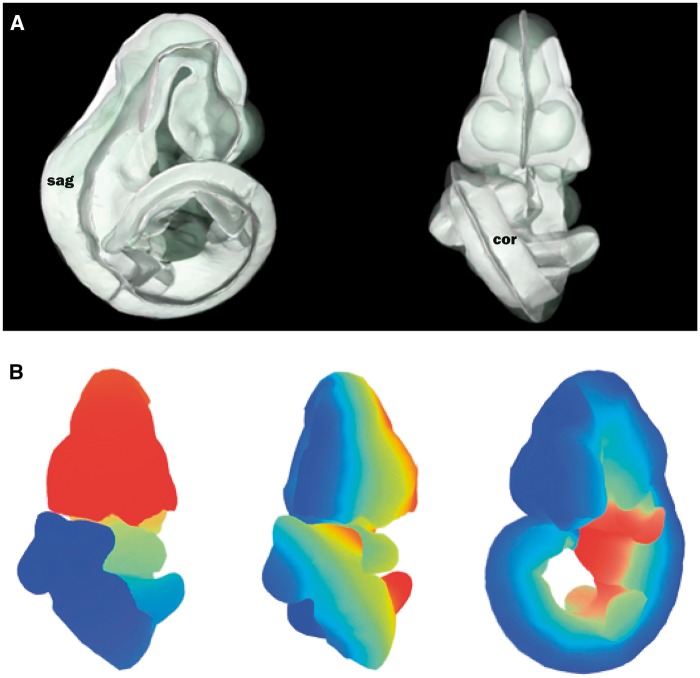
Defining axial planes on the 3D mouse embryo model. **(A)** Reverse spatial warping allowed axial planes to be defined on the straight mouse embryo model. Sagittal and coronal planes from this reverse transform are shown in the context of the original 3D mouse embryo model. sag, sagittal plane; cor, coronal plane. **(B)** Colourmaps from red to blue used to demonstrate A–P (left), L–R (middle) and V–D (right) axes in the original, curled embryo model. These axes represent the anatomical coordinates of the embryo model.

The straight mouse embryo model was built using the midline signalling molecule *Shh* and anatomical knowledge so that the Cartesian *z-*, *y-* and *x-*axes in the straight embryo correspond to the anatomical A–P, D–V and L–R axes in the embryo. The expression patterns of the straight embryo can be seen to correspond to those of the original curved embryo as shown in Figure 2A and B. The *Shh* expression pattern in the midline of the A–P axis is clearly visible in the dorsal views of the original and straightened embryo models; *Shh* is additionally expressed in the ZPA in the posterior region of the limb buds (11), and this enabled accurate identification of the A–P axis in the developing forelimb and hindlimb buds. In the straight mouse, D–V and L–R axes were defined orthogonally to the *Shh* pattern. Through applying the inverse transform to *z-x*, *y-z* and *x-y* planes of the straight embryo model, these planes were observed to map to the sagittal, coronal and transverse cardinal planes of the original embryo reference space (Figure 3A). Axes were also visualized by means of a colourmap (Figure 3B). We used anatomical features and the *Shh* expression pattern to verify an accurate correspondence between straight and curved models. As part of this visual inspection process, D-V axial planes were further validated by ensuring that they passed symmetrically though paired anatomical components of both the straight mouse embryo and the original embryo reference space, including optic cups, nasal placodes, maxillary and mandibular components of the first branchial arch, forelimb buds, and hindlimb buds. By virtue of this curation we ensured that the straight mouse embryo was an accurate representation of 3D embryo space.

## Novel visualization

We have explored the use of the straight mouse embryo to visualize spatial data using spatially mapped gene expression patterns of *Fgf8*, *Fgf9*, *Fgf10* and *Fgf20*. These genes show distinct, but overlapping gene expression patterns, which are apparent in both the original curved and straight mouse embryos. Using a metric that calculates the number of voxels with mapped expression per unit volume, the straight mouse representation clearly shows that the *Fgf* gene expression patterns are organized sequentially in the craniofacial region, with *Fgf8* showing a peak of expression in the anterior-most compartment of the craniofacial region, and peaks of *Fgf9*, *Fgf10* and *Fgf20* expression being observed in more posterior compartments (Figure 2C). Peaks of *Fgf8* and *Fgf10* expression were additionally observed in the A–P compartments that included the forelimb and hindlimb buds. A profile of spatial expression along the A–P axis was easily generated through use of Cartesian coordinates in the straight mouse model (Figure 2C).

To further demonstrate how the straight mouse embryo anatomical coordinate system can be used to analyse gene expression data, we used the straight mouse embryo model to describe spatial relationships in terms of D–V, L–R, proximal–distal (P–D) and medial–lateral (M–L) locations (Table 1). All of these directional and locational terms could be defined in the straight, but not the original curled mouse model in the context of anatomical regions delineated in the 3D model. A subset of paired spatial relationships between *Shh* and *Fgf* gene expression patterns that were defined in the context of the straight mouse embryo model is listed in Table 1. We consider these paired spatial relationships to be useful from a database perspective, in that they would allow a list of genes to be ordered in terms of sequential distribution along A–P, D–V or L–R axes. Of interest, there was an observed ambiguity in the L–R relationships between *Shh* and *Fgf10* in the hindbrain region, whereby Shh was both *left_of* and *right_of Fgf10*, and *vice versa*. However on closer inspection this could be seen to reflect the M-L relationship between *Shh* and *Fgf10*, whereby the midline signalling molecule *Shh* is expressed *medial_to Fgf10* that is laterally expressed in both left and right hindbrain.

**Table 1.** Paired spatial relationships derived from straight mouse embryo model

Term	Description	Paired spatial relationships
A–P	Anterior is the head or cranial end, posterior is the tail or caudal end.	*Fgf8* is *anterior_to Fgf20* in the craniofacial region.
		*Fgf20* is *posterior_to Fgf8* in the craniofacial region.
D–V	Dorsal refers to the backbone side, ventral refers to the belly side.	*Fgf10* is *dorsal_to Shh* in the midbrain region.
		*Shh* is *ventral_to Fgf10* in the midbrain region.
L–R	Left-right as defined looking from dorsal to ventral.	*Fgf10* is *left_of Shh* in the hindbrain region.
		*Shh* is *right_of Fgf10* in the hindbrain region.
		*Fgf10* is *right _of Shh* in the hindbrain region.
		*Shh* is *left_of Fgf10* in the hindbrain region.
M–L	Relative positional terms, medial structures are relatively close to the midline, lateral are further to the side.	*Fgf10* is *lateral_to Shh* in the hindbrain region.
		*Shh* is *medial_to Fgf10* in the hindbrain region.
P–D	Relative positional terms applicable to appendicular structures. Proximal is closer to the centre of the body and distal is further away.	*Fgf10* is *proximal_to Fgf8* in the left forelimb bud.
		*Fgf8* is *distal_to Fgf10* in the left forelimb bud.

## Discussion

The anatomical coordinate system that we have outlined here—‘*the straight mouse’*—has allowed us to define the major axes of the embryo such that we are now able to computationally define the midline, and to define patterns in relation to the midline in terms of A–P, D–V and L–R coordinates. A similar ‘straightening’ process has been demonstrated previously in the nematode *Caenorhabditiselegans* (12), but to our knowledge this is the first time this process has been applied to a vertebrate embryo model which is inherently more complex. To ensure that we correctly defined the A–P axis in the mouse embryo reference space, we used a *Shh* labelled E10.5 mouse embryo to delineate the axial midline of the head, trunk and tail and in this way we could validate that the midline was mapped accurately onto the ‘straight mouse’ model. The axial pattern of *Shh* appears to be an evolutionary innovation unique to vertebrates, as *Shh* is not expressed in the notochord of simpler chordates such as *Ciona interstinalis* (13). In vertebrate embryos, *Shh* is expressed in the notochord and floor plate (7)—both midline structures—and additionally in the head region where it extends rostrally in the brain as far as the diencephalon (14). Furthermore, *Shh* is expressed in the ZPA in the posterior region of the limb bud (11), and this expression pattern, spatially mapped onto the straight model, enabled us to accurately define the A–P axis in the developing forelimb and hindlimb buds (Figure 2B). Once we had defined the anatomical coordinates in the ‘straight embryo’ model, it was straightforward to introduce this coordinate system into the original model using a reverse spatial transform.

This anatomical coordinate system enables analysis of the distribution of spatially mapped gene expression patterns. Spatial mapping of gene expression patterns is utilized by the EMAGE gene expression database and is a powerful means of measuring coexpression in biological systems. This is an important method as gene expression patterns often do not conform to defined anatomical boundaries, and consequently text annotation of complex patterns must be supported by spatial annotation. However, there are limitations to the computational measures that can be applied to spatially mapped data. Specifically, by focusing on voxel-space (*xyz*) coordinates, the computational measures that are most appropriate to scoring co-expression, such as the ‘Jaccard index/Dice coefficient’, do not address, e.g. the A–P and proximo-distal expression of the *Hox* genes (reviewed in 15). The anatomical coordinate system that we introduce in the context of *Fgf* gene expression resolves this issue, and enables patterning processes, as assayed through expression analysis of developmentally regulated genes, to be understood in an axial framework. This would enable e.g. the ranking of gene expression patterns along the A–P, D–V or L–R axes. We explored this concept by using the straight mouse embryo model to describe spatial relationships between *Shh* and *Fgf* gene expression patterns. In the straight mouse embryo model, we could define paired spatial relationships for anatomical compartments, e.g. *Fgf10* is *dorsal_to Shh* in the midbrain region; *Shh* is *ventral_to Fgf10* in the midbrain region. We foresee these paired spatial relationships as particularly useful for ordering gene expression patterns in terms of sequential distribution along A–P, D–V or L–R axes. This is of great interest from a biomedical perspective as it may offer one method by which database queries could be used to explore the architectonic nature of gene expression. These architectonic relationships are crucially important as they may underlie the basis of congenital abnormalities. In this respect it is noteworthy that *Fgf* and *Shh* signalling is critically important in craniofacial development, and that mutations in key *Fgf* and *Shh* signalling genes cause a spectrum of craniofacial midline abnormalities such as clft lip, cleft palate, and frontonasal dysplasia (16–19). The straight mouse model that we have developed represents one method through which spatially mapped gene expression patterns can be structured and implemented into database queries.

An additional use of the anatomical coordinate system is as a means of evaluating the metameric nature of the vertebrate embryo body plan. Visible in the trunk and tail of the developing embryo are the segmented transitional tissues known as somites, that will subsequently split to form the dermatome (dermis), myotome (skeletal muscle), syndetome (tendons and cartilage), and sclerotome (bone). The somites additionally pattern the peripheral nervous system through specifying the migration paths of neural crest cells and the axons of spinal nerves. In the head region the cranial nerves are organized along the A–P axis with the anterior-most cranial nerves (cranial nerves 0 and I) terminating in the olfactory cavity and the olfactory organs (20) and the most posterior cranial nerves providing motor innervation for muscles of the neck (cranial nerve XI) and the tongue (cranial nerve XII). A more controversial segmented organization of the developing head has additionally been suggested. Kingsbury (21) hypothesized that the head is segmentally organized, with each head segment containing a neuromere plus a pair of dorsal and ventral cranial nerves. The value of the neuromeric hypothesis is that it may provide insight into iterative processes that may underlie brain development. More recently, developmental neurobiologists have lent support to the neuromeric hypothesis by using gene expression patterns in the developing brain as molecular anatomical profiles that enable otherwize cryptic anatomical boundaries to be delineated (reviewed in 22). In this respect, the anatomical coordinates-based distribution of gene expression that we showcase in the context of *Fgf* gene expression in the straight mouse embryo may greatly assist in the identification of cryptic boundaries in the developing mouse embryo, and offer an axial framework that could be used to evaluate the neuromeric hypothesis.

The anatomical coordinate spatial framework may additionally enable text-mining of spatially mapped data. In this respect, the Biological Spatial Ontology (BSPO) has recently been developed as a means of representing spatial concepts, anatomical axes, gradients, regions, planes, sides and surfaces using a vocabulary (23). The BSPO is currently used by projects that require standardized anatomical descriptors for phenotype annotation and ontology integration, and is used to provide a source of anatomical location descriptors for logically defining anatomical entity classes in anatomy ontologies. The anatomical coordinate embryo reference space that we describe here lends itself quite naturally to the BSPO classes and relations, and these could be used to text-mine datasets that are spatially mapped onto an embryo reference model. An interesting dataset to explore using this method would be the Eurexpress transcriptome-wide atlas of mouse embryo gene expression (24). The eMouseAtlas Project has spatially mapped 14K Eurexpress *section in situ* hybridization gene expression patterns onto an E14.5 (TS 23) embryo model, and these patterns can be ranked by spatial similarity (i.e. coexpression) and/or retrieved by spatial query using tools made publicly available through EMAGE (25, 26). Using a text-mining approach, it may be possible to use BSPO ontology terms such as ‘adjacent_to, dorsal_to’, ‘orthogonal_to’, as queries on the spatially mapped Eurexpress dataset. To develop this type of query capability, our future work will focus on developing a similar type of anatomical coordinate spatial framework for our later stage embryo models.

In summary, we have used spatial warping and marker gene expression patterns to develop an anatomical coordinate system for the mouse embryo. To develop an anatomical coordinate spatial framework, we straightened a 3D mouse embryo model through deformation of 3D volumetric embryo space, defined the A–P, D–V and L–R axes on the ‘straight mouse’ model, and introduced this coordinate system into the original model using a reverse ‘spatial warp’ transform. In this analysis, we have pioneered the use of the ‘straight mouse’ embryo as a means of generating anatomical coordinates for a TS 17 mid-gestation mouse embryo model. Through applying this process to EMAP mouse embryo models at other stages of development, we could provide a similar anatomical coordinate framework for spatially mapped gene expression patterns at earlier and later stages of development. We see great potential of this anatomical coordinate reference space in summarizing spatially mapped multimodal mouse embryo data, which can be otherwise complex and very laborious to describe.

## References

[1] Thompson D.W. (1917) On Growth and Form,1st edn Cambridge University Press, Cambridge.

[2] French V., Bryant P.J., Bryant S.V. (1976) Pattern regulation in epimorphic fields. Science, 193, 969–981. 948762 10.1126/science.948762

[3] Bryant S.V., French V., Bryant P.J. (1981) Distal regeneration and symmetry. Science, 212, 993–1002. 17779956 10.1126/science.212.4498.993

[4] Paxinos G., Watson C. (1997) The Rat Brain in Stereotaxic Coordinates. Academic Press, San Diego, USA.

[5] Baldock R., Burger A. (2008) Anatomy ontologies: linking names to places in biology. In: Burger,A., Davidson,D., Baldock,R. (eds). *Anatomy Ontologies for Bioinformatics: Principles and Practice* 1st edn. Springer-Verlag London. pp. 197–212.

[6] Hill B., Baldock R.A. (2015) Constrained distance transforms for spatial atlas registration. BMC Bioinformatics, 2015, 16, 90. 25887037 10.1186/s12859-015-0504-5 PMC4374577

[7] Martí E., Takada R., Bumcrot D.A. (1995) Distribution of Sonic hedgehog peptides in the developing chick and mouse embryo. Development, 121, 2537–2547. 7671817 10.1242/dev.121.8.2537

[8] Sharpe J., Ahlgren U., Perry P. (2002) Optical projection tomography as a tool for 3D microscopy and gene expression studies. Science, 296, 541–545. 11964482 10.1126/science.1068206

[9] Theiler K. (1972) The House Mouse: Atlas of Embryonic Development, 1st edn New York: Springer-Verlag.

[10] Armit C., Venkataraman S., Richardson L. (2012) eMouseAtlas, EMAGE, and the spatial dimension of the transcriptome. Mammalian Genome, 23, 514–524. 22847374 10.1007/s00335-012-9407-1 PMC3463796

[11] Riddle R.D., Johnson R.L., Laufer E., Tabin C. (1993) Sonic hedgehog mediates the polarizing activity of the ZPA. Cell, 75, 1401–1416. 8269518 10.1016/0092-8674(93)90626-2

[12] Peng H., Long F., Liu X. (2008) Straightening Caenorhabditis elegans images. Bioinformatics, 24, 234–242. 18025002 10.1093/bioinformatics/btm569 PMC2940239

[13] Takatori N., Satou Y., Satoh N. (2002) Expression of hedgehog genes in Ciona intestinalis embryos. Mech Dev, 116, 235–238. 12128232 10.1016/s0925-4773(02)00150-8

[14] Lim Y., Golden J.A. (2007) Patterning the developing diencephalon. Brain Res Rev, 53, 17–26. 16876871 10.1016/j.brainresrev.2006.06.004

[15] Pearson J.C., Lemons D., McGinnis W. (2005) Modulating Hox gene functions during animal body patterning. Nat Rev Genet, 6, 893–904. 16341070 10.1038/nrg1726

[16] Brewer J.R., Molotkov A., Mazot P. (2015) Fgfr1 regulates development through the combinatorial use of signaling proteins. Genes Dev, 29, 1863–1874. 26341559 10.1101/gad.264994.115 PMC4573858

[17] Jin Y.R., Han X.H., Taketo M.M., Yoon J.K. (2012) Wnt9b-dependent FGF signaling is crucial for outgrowth of the nasal and maxillary processes during upper jaw and lip development. Development, 139, 1821–1830. 22461561 10.1242/dev.075796 PMC3328181

[18] Trumpp A., Depew M.J., Rubenstein J.L. (1999) Cre-mediated gene inactivation demonstrates that FGF8 is required for cell survival and patterning of the first branchial arch. Genes Dev, 13, 3136–3148. 10601039 10.1101/gad.13.23.3136 PMC317178

[19] Kurosaka H., Iulianella A., Williams T., Trainor P.A. (2014) Disrupting hedgehog and WNT signaling interactions promotes cleft lip pathogenesis. J Clin Invest, 124, 1660–1671. 24590292 10.1172/JCI72688 PMC3973078

[20] Fuller G.N., Burger P.C. (1990) Nervus terminalis (cranial nerve zero) in the adult human. Clin Neuropathol, 9, 279–283. 2286018

[21] Kingsbury B.F. (1926) Branchiomerism and the theory of head segmentation. J. Morphol, 42, 83–109.

[22] Martínez S., Puelles E., Puelles L., Echevarria D. (2012) Molecular regionalization of the developing neural tube In: Watson G., Paxinos G., Puelles L. (eds). The Mouse Nervous System. Elsevier, Amsterdam, pp. 2–18.

[23] Dahdul W.M., Cui H., Mabee P.M. (2014) Nose to tail, roots to shoots: spatial descriptors for phenotypic diversity in the Biological Spatial Ontology. J Biomed Semantics, 5, 34. 25140222 10.1186/2041-1480-5-34 PMC4137724

[24] Diez-Roux G., Banfi S., Sultan M. (2011) A high-resolution anatomical atlas of the transcriptome in the mouse embryo. PLoS Biol, 9, e1000582. 21267068 10.1371/journal.pbio.1000582 PMC3022534

[25] Armit C., Richardson L., Hill B. (2015) eMouseAtlas informatics: embryo atlas and gene expression database. Mamm Genome, 26, 431–440. 26296321 10.1007/s00335-015-9596-5 PMC4602050

[26] Armit C., Richardson L. (2016) Digital graphical resources and developmental anatomy in the mouse. In: Baldock,R., Bard,J., Davidson,D.R., Morriss-Kay,G. (eds). *Kaufman’s Atlas of Mouse Development Supplement: With Coronal Sections* 1st edn. Academic Press, Elsevier. pp. 295–206.

